# The good, the bad and the ugly of transposable elements annotation tools

**DOI:** 10.1590/1678-4685-GMB-2023-0138

**Published:** 2024-02-19

**Authors:** Elgion L. S. Loreto, Elverson S. de Melo, Gabriel L. Wallau, Tiago M. F. F. Gomes

**Affiliations:** 1 Universidade Federal do Rio Grande do Sul, Programa de Pós-Graduação em Genética e Biologia Molecular, Porto Alegre, RS, Brazil.; 2 Fundação Oswaldo Cruz, Instituto Aggeu Magalhães, Departamento de Entomologia, Recife, PE, Brazil.; 3 Universidade Federal de Santa Maria, Departamento de Bioquímica e Biologia Molecular, Santa Maria, RS, Brazil.

**Keywords:** Transposable elements, bioinformatics, annotation, classification

## Abstract

Transposable elements are repetitive and mobile DNA segments that can be found in virtually all organisms investigated to date. Their complex structure and variable nature are particularly challenging from the genomic annotation point of view. Many softwares have been developed to automate and facilitate TEs annotation at the genomic level, but they are highly heterogeneous regarding documentation, usability and methods. In this review, we revisited the existing software for TE genomic annotation, concentrating on the most often used ones, the methodologies they apply, and usability. Building on the state of the art of TE annotation software we propose best practices and highlight the strengths and weaknesses from the available solutions.

## Introduction

Transposable elements (TEs) are mobile genetic elements found in nearly every eukaryotic organism studied to date. As the name implies, these elements use the host molecular machinery to code their protein for mobilization. TEs are repetitive and sometimes fragmented, may be found within other TEs or protein-coding genes, and exhibit a wide range of structural, sequence-length, diversity and distribution across species. TEs constitute a significant portion of the genomes of many eukaryotic organisms, as for instance, 45% of the human genome and 85% of the maize genome (Saleh *et al.*, 2019; Stitzer *et al.*, 2021; Hayward and Gilbert, 2022). The method of transposition used by TEs varies depending on the TE class. Class I elements transpose via an RNA intermediate using a reverse transcriptase in what is known as “copy-and-paste” transposition; class II elements transpose via a DNA intermediate, with the majority of elements, called TIR (terminal inverted repeats) elements, in this class using “cut-and-paste” mechanism, which is performed by enzymes known as transposases (Wells and Feschotte, 2020). TEs are yet subdivided in order, superfamily, family and subfamily (Wicker *et al.*, 2007; Makałowski *et al.*, 2019). In some species, *e.g. Homo sapiens*, despite having a high number of TEs, few are known to be active, such as *Alu*, *L1*, SVA (SINE-VNTR-Alu) and non-LTR class I elements (Ali *et al.*, 2021; Autio *et al.*, 2021). Furthermore, not all elements have the required machinery to transpose, and those lacking it are referred to as non-autonomous elements, relying on autonomous elements, which have the necessary enzymes to transpose. This is illustrated by the previously mentioned elements *L1* and *Alu* in humans, with the latter relying on the former to insert into a new genomic loci (Burns, 2020; Chesnokova *et al.*, 2022).

Using bioinformatics to find and characterize TEs is particularly challenging like putting together a puzzle with multiple copies of the same or very similar pieces, each with its own place, some shredded or with holes in it, and other pieces glued together with another piece. Therefore, choosing the right tools to find and classify TEs in genomes is a difficult task as there is currently no single tool that can thoroughly fulfill this effort on its own. Similarity-based, structure/motif pattern-matching, *de novo* prediction, or a workflow combining different methods are the approaches used by TEs annotation software, each with a trade-off between its strengths and weaknesses that need to be equated when choosing a program, that is, the good and the bad algorithmically speaking. There are two other frequently encountered software issues found by researchers that we consider to be the “ugly” part of the TE annotation software story: user friendliness and application development state.

Many of the most commonly used applications are not well maintained, failing to keep up with operating system updates or advances in the programming languages in which they are written, resulting in difficulties in installation due to the obsolete dependencies required by the software (Mangul *et al.*, 2019). The problem of finding and installing the correct package versions can be overcome by using programs to create virtual environments or “containers”. However, this does not guarantee that the required dependency versions will be available or that it will be easier to install. Another option is to compile either the software or its dependencies from the source code, which may result in a time-consuming snowball effect of finding software dependencies, all of which must be compatible with the operating system used.

To complete the task of installing and using the software, the human side must be considered. It necessitates skills that, depending on the researcher’s background, may outweigh his or her knowledge or willingness to use the software. In line with this, not all software has a complete and clear documentation on how to run them and what the available options mean.

Herein, we bring to light the good, the bad and the ugly sides of using bioinformatics tools for genomic annotation of transposable elements. We addressed the most commonly used softwares, how to distinguish between methods, and what can be done to advance the current state-of-the-art on the subject.

## Methods and software for TE annotation

The process of detecting a TE sequence in a genome, classifying it, and identifying its coordinates, *i.e.* the start and end of a sequence, in a chromosome or contigs is referred to as TE annotation. The repetitiveness of TEs, the number of very similar or degraded copies, and the presence of nested elements are some of the challenges faced by TE annotation software. Tools designed to annotate TEs may use sequence similarity, the presence of structural elements, such as long terminal repeats (LTR) or terminal inverted repeats (TIR), a *de novo* or a combination of these approaches to accomplish this task. 

Table 1 summarizes the main features of the softwares used for TE annotation and classification, such as the release year, method for TE characterization, the software development status, *i.e*., whether it is still receiving updates, improvements, or developer support, and other aspects such as the operating system required to run the software if it is downloadable version.

**Table 1 -:** Summary of the key features of tools used to annotate or classify TEs.

Software	Year	Type	Method	Development status	Presentation	Operating system	Installation	External dependencies	Alternative installation
CENSOR	1996	Annotator	Similarity	Maintained*	Web/Downloadable	-	-	No	-
ClassifyTE	2021	Classifier	Machine learning	Maintened	Downloadable	Unix	Executable script	Yes	Venv
DAWGPAWS	2009	Annotator	Combined	Not maintained	Downloadable	Unix	Executable script	Yes	-
DeepTE	2020	Classifier	Machine learning	Maintained	Downloadable	Unix	Executable script	Yes	Venv
EarlGrey	2022	Annotator	Combined	Maintained	Downloadable	Linux	Executable script	Yes	Venv/Container
EDTA	2019	Annotator	*De novo*	Maintened	Downloadable	Linux	Executable script	Yes	Venv/Container
LTR annotator	2015	Annotator	*De novo*	Not maintained	Downloadable	Linux	Executable script	Yes	-
LTR classifier	2016	Classifier	Library-based	Maintained	Web	-	-	-	-
LTR_finder	2007	Annotator	Structure	Not maintained	Downloadable	Linux	Source code	No	-
MITE-hunter	2010	Annotator	Structure	Not maintained	Downloadable	Linux	Executable script	No	-
MITE-tracker	2018	Annotator	Structure	Maintained	Downloadable	Unix/Windows	Executable script	Yes	-
PASTEC	2014	Classifier	Library-based	Maintained	Downloadable	Linux	Executable script	Yes	Container
reasonaTE	2022	Annotator	Combined	Maintained	Downloadable	Linux	Executable script	Yes	Venv
REPCLASS	2015	Classifier	Library-based	Not maintained	Downloadable	Linux	Executable script	Yes	-
RepeatClassifier	2020	Classifier	Library-based	Maintaned	Downloadable	Linux	Executable script	-	Venv/Container
RepeatMasker	1997	Annotator	Similarity	Maintained	Web/Downloadable	Linux	Executable script	Yes	Venv/Container
RepeatModeler	2008/2020	Annotator	*De novo*	Maintained	Downloadable	Linux	Executable script	Yes	Venv/Container
REPET	2011	Annotator	Combined	Maintained	Downloadable	Linux	Executable script	Yes	Container
RFSB	2022	Classifier	Machine learning	Maintained	Downloadable	Linux	Executable script	Yes	Venv
RFSB	2022	Classifier	Machine learning	Maintaned	Downloadable	Linux	Executable script	Yes	Venv
RTclass1	2010	Classifier	Library-based	Maintained	Web/Downloadable	Linux	Executable script	Yes	-
TERL	2020	Classifier	Machine learning	Maintained	Downloadable	Unix	Executable script	No	Venv
TEsorter	2022	Classifier	Library-based	Maintained	Downloadable	Unix	Executable script	Yes	Venv
TIR-learner	2019	Annotator	Combined	Maintained	Downloadable	Linux	Executable script	Yes	-
TIRmite	2017	Annotator	Structure	Maintained	Downloadable	Linux	Executable script	Yes	Venv

Executable script: the program does not need to be compiled from source code; Source code: program needs to be compiled to install; Venv: program can be installed using a virtual environment; Container: program can be installed as a container using tools such as Docker or Singularity. Linux: program needs to be installed in a Linux based operating system (OS); Unix: a Unix-based operating system such as MacOS or a Linux-based OS; External dependencies: tools mandatory to run the main program that are not installed in the main program installation; *just the web version appears to be maintained.

## Similarity-based

**Figure 1 -:**
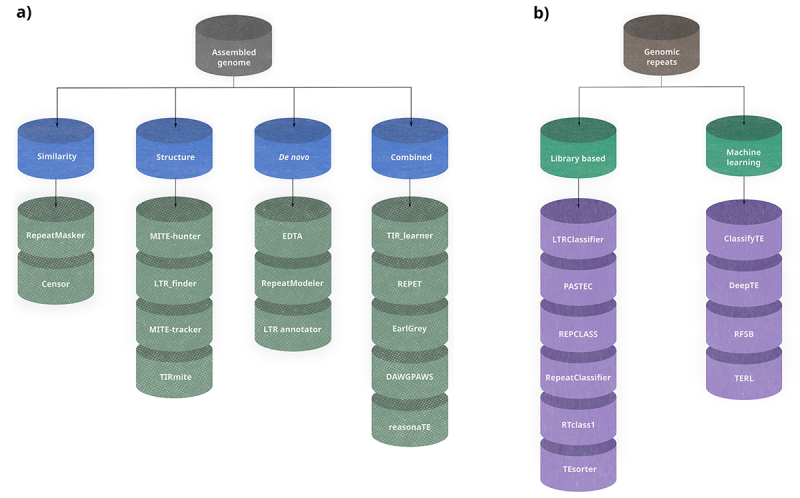
Schematic representation of some software available for TE annotation (a) and classification (b) based on the method for TE detection.

The most used method for characterizing TE sequences employ similarity-based methods (Zielezinski *et al.*, 2017; Carey *et al.*, 2021), many times wrongly named homology-based. Homology is a qualitative term that establishes the existence or not of an evolutionary relationship, whereas similarity is a quantitative term referring to the percentage of similarity between two sequences, which might lead to the conclusion of homology due to high similarity. Furthermore, there can be homologous sequences of low similarity and sequences of high similarity that are the result of convergent evolution. (Reeck *et al.*, 1987; Pearson, 2013). The similarity method is used by RepeatMasker (Smit *et al.*, 2013) and CENSOR (Kohany *et al.*, 2006), two of the most well-known and widely used tools for masking repetitive sequences (Figure 1 a ). Similarity-based searches have high specificity and accuracy, making it useful for detecting conserved regions of related sequences, single nucleotide polymorphisms, and indels. Disadvantages are their heavy computational demand, it may not work well with highly divergent sequences (Mitchell-White *et al.*, 2021; Hubley *et al.*, 2022; Hénault *et al.*, 2023), can generate false positives when working with repetitive sequences as TEs, due to incomplete sequences, sequencing errors, permissive parameters not suited for repetitive sequences, and databases with low quality or redundant sequences resulting in spurious alignments (Markova-Raina and Petrov, 2011; Fujimoto *et al.*, 2016; Choe *et al.*, 2023), and are limited to known sequences, *i.e.*, do not allow the discovery of completely new TEs with no similarity to know TEs from databases.

RepeatMasker searches genomic data for interspersed repeats and low complexity DNA sequences, by default using genomic chunks as queries against the Dfam database, including Hidden Markov Models profiles and consensus sequences (Storer *et al.*, 2021), but it is also possible to use a Repbase-like formatted custom library instead. RepeatMasker is written in Perl, an interpreted programming language, meaning it does not need to be compiled from source, it includes installation instructions, basic usage and a detailed program manual with all of the information needed regarding all parameters. It can be installed from the bioconda channel in a conda virtual environment. RepeatMasker is still maintained, updated, and launches newer releases on its website. It is an open-source software available for download at https://www.repeatmasker.org/ or https://github.com/rmhubley/RepeatMasker.

CENSOR compares nucleotide or amino acid sequences to known repeats using WU-BLAST (in newer paid versions there is an option to use BLAST instead), and can compare sequences of DNA-DNA or DNA-protein. CENSOR is available as a web-based service or standalone program to be used in UNIX systems. The web version uses the REPBASE database, which requires a paid subscription to download since 2018. The standalone version available for download (at https://www.girinst.org/*dow*nloads/software/censor) was last updated in 2016, has a short description on how to use it and no manual describing the options.

## Structure-based

Tools that search for structure in sequences, also called signature-based, can discover catalytic sites and functional protein sites or sequence structures as TIRs and LTRs at DNA level (Storer *et al.*, 2022). They can also be used to improve similarity-based alignment results. This method is limited by the availability of known sequence structures and does not work well with highly variable regions or homologs that are highly divergent. Two of the most used softwares using this method are LTR_finder (Xu and Wang, 2007) and MITE-hunter (Han and Wessler, 2010), as well as other tools such as MITE-tracker (Crescente *et al.*, 2018) and TIRmite (found at https://github.com/Adamtaranto/TIRmite).

LTR_finder identifies full-length LTR elements in genomic data by searching possible exactly matching pairs at the 5’ and 3’ end of sequences, selecting the pairs based on a specified distance between them, calculates the similarity between regions using global alignment and adjusts the near-end boundaries using the Smith-Waterman algorithm. It is presented both as a web-server and a standalone version for UNIX systems. The latter is written in C and C++ and must be compiled from the source code. It is also dependent on Perl. The manual makes no mention of dependency versions or the requirement to install the Perl module GD, which is required for bitmap handling. The LTR_finder repository on github (https://github.com/xzhub/LTR_Finder) is no longer maintained and the webserver was not available (http://tlife.fudan.edu.cn/ltr_finder/), at the moment of writing review.

MITE-hunter is a program that searches for miniature inverted-repeat transposable elements (MITEs), which are short non-autonomous Class II elements found in plants and animals. MITE-hunter is written in Perl and is intended to run on UNIX systems. It first identifies candidates based on the presence or absence of TIRs and target site duplications (TSDs), then performs an all-by-all BLASTN comparison to filter false positives and clusters selected sequences. A multiple sequence alignment is performed to generate consensus sequences, which are then categorized into families. It can be downloaded on http://target.iplantcollaborative.org/mite_hunter.html, but does not appear to be in development any longer, as the last update on its github page (https://github.com/jburnette/MITE-Hunter) was in 2010. MITE-hunter depends on NCBI BLAST, Muscle, mDust and the Perl programming language to be installed and used. The manual makes no mention of the dependencies versions.

## De novo

The *de novo* method does not require a reference database to find TEs, which is useful when working with newly sequenced genomes. Conversely, it can produce unreliable results due to sequencing or assembling errors, and because there are no curated sequences as reference to validate the results. It usually works by performing an all-by-all sequence comparison followed by sequence clustering or by directly applying clustering methods to reads that will be downsampled or filtered (Storer *et al.*, 2022). RepeatModeler (Smit and Hubley, 2008), EDTA (Ou *et al.*, 2019) and LTR annotator (You *et al.*, 2015) are some examples of tools using this method, being RepeatModeler and EDTA two of the most used in the literature.

RepeatModeler is a pipeline for *de novo* TE identification that aims to produce a reliable and consistent TE library of consensus sequences of unique TE families. It uses Recon for repeat discovery, which employs a sensitive alignment approach and is well suited to discovering old TE families, and RepeatScout, which is faster and detects the most abundant and younger families more easily. RepeatModeler is mostly written in Perl, having a complete and detailed manual on how to install and run it, with all of its dependencies clearly specified with the necessary versions. It is still maintained and is available at https://github.com/Dfam-consortium/RepeatModeler or http://www.repeatmasker.org/RepeatModeler/. The newer version RepeatModeler2 (Flynn *et al.*, 2020) integrates a structure discovery step of LTR elements to improve the discovery of elements of this class. 

EDTA is a package designed for *de novo* TE annotation that aims to generate a high-quality non-redundant TE library for whole sequenced genomes. It was developed by benchmarking many TE tools using a manually curated rice TE library, and selecting the most performant ones to be part of the TE annotation pipeline, which includes LTRharvest, a parallel version of LTR_FINDER, LTR_retriever, GRF, TIR-Learner, HelitronScanner, and RepeatModeler2. EDTA is written using Perl, Python and shell script, and can be installed using a conda virtual environment, singularity or docker containers. Its manual contains detailed descriptions on how to install and run the program, as well as information on the input and output files. It is still maintained and updated, being found at https://github.com/oushujun/EDTA. It can also be used to test new TE annotation methods or TE libraries using the rice genome, according to the authors of EDTA. The input FASTA sequence identifiers (IDs) must be at most 13 characters long, and many non-alphanumeric characters are not permitted; otherwise, the program execution is terminated. There is no tool or script included with the package to edit the invalid IDs, leaving it up to the user to do so.

## Combined approaches

Because TEs are such complex elements with so many features to consider in order to correctly annotate them, the scientific community has agreed that a combination of *de novo*, similarity, and structure-based approaches is the best strategy for a more careful and accurate characterization of TEs. TIR-learner (Su *et al.*, 2019), REPET (Flutre *et al.*, 2011), reasonaTE - part of TransposonUltimate (Riehl *et al.*, 2022), DAWGPAWS (Estill and Bennetzen, 2009) and Earl Grey (Baril *et al.*, 2022) are examples of such tools.

TIR-learner is a tool developed to detect TIRs primarily in plant genomes and is available at https://github.com/WeijiaSu/TIR-element-annotation. It uses a pipeline of combining similarity and structure approaches with a *de novo* structure screening, which uses a machine learning algorithm to classify sequences into five TIR superfamilies. Next, it removes overlaps by comparing the outputs of each method, resulting in a library of TIR-elements. It is written in Python and shell script, and it is dependent on the software Generic Repeat Finder (GRF) and BLAST+. It includes a simple and straightforward manual for installing and running the software. There is no mention of specific version dependencies. Its most recent version is 1.14, which was updated in 2019 with newer unresolved github issues.

REPET is a software suite that uses two main pipelines to annotate TEs at the genomic scale: TEdenovo and TEannot. The former compares a genome to itself using BLASTER and then clusters the resulting matches using GROUPER, RECON, and PILER. For each cluster, a multiple sequence alignment is performed in order to construct a consensus sequence and then classify it. After that, TEannot combines multiple programs to reconstruct intact TE copies and filter out fragmented copies and false-positives. REPET is written in C++ and Python to be used in Linux-based systems, it depends on several external programs, with some dependency versions being deprecated or not yet maintained upstream, such as the required Python version (version 2.x). To help address those issues, there is a docker version. The REPET manual has detailed information about software versions, installation and usage. It is still maintained, with recent updates on its containerized version including PFAM database and a newly added eukaryotic rRNA database. The REPET package and its instructions can be found at http://urgi.versailles.inra.fr/Tools/REPET.

## Classifiers

Following the step of generating a series of TE consensus, the newly created library must be classified, which will give those sequences some taxonomical meaning. Although many TE annotation pipelines rely on some sort of classification mechanism (Flutre *et al.*, 2011; Flynn *et al.*, 2020; Riehl *et al.*, 2022), this mechanism does not always follow a classification scheme adopted by a research group, or provide the level of detail desired by the researcher. Furthermore, different classifiers generate predictions using different databases as a source of comparison. The distribution of TE types in a database, as well as the divergence between the species under study and the species present in the database, will have a direct impact on the classification quality, because there is a loss of TE identification when very divergent reference sequences are used (Bell *et al.*, 2022). It is also known that different classification methods have varying accuracies, with some better classifying specific groups of TEs than others (Hoede *et al.*, 2014; Monat *et al.*, 2016; Zhang *et al.*, 2022). As a result, it is frequently necessary to apply multiple classification methods to a newly created library in order to resolve ambiguities in more divergent consensus (Melo and Wallau, 2020).

In recent years, TE classification mechanisms have evolved significantly. In general, they can be divided into two large groups (Figure 1b): I) programs that employ traditional approaches, such as the use of various types of blasts and search algorithms for protein domains like HMMER. including REPCLASS (Feschotte *et al.*, 2009), PASTEC (Hoede *et al.*, 2014), RepeatClassifier (a classification program from RepeatModeler 2), LTRclassifier (Monat *et al.*, 2016), TEsorter (Zhang *et al.*, 2022) and RTclass1 (Kapitonov *et al.*, 2009); II) programs that use machine learning algorithms, including TEclass (Abrusán *et al.*, 2009), DeepTE (Yan *et al.*, 2020), ClassifyTE (Panta *et al.*, 2021), RFSB (part of TransposonUltimate) and TERL (da Cruz *et al.*, 2021).

One of the most cited classifiers is PASTEC. It is part of the REPET pipeline and thus has the same set of manuals, whether it is installed alongside the main package or used within a container provided by the developers. PASTEC searches sequences for structural features such as TIRs or LTRs, as well as the presence of SSRs, ORFs, and poly(A) tails. This program also searches for sequence similarity against Repbase sequences and Pfam domains. One of the most interesting aspects of PASTEC is its user-friendly output, which includes a tabular file with a classification combined with a confidence index for each sequence, as well as lists of structural characteristics, protein domains, and blast matches against Repbase. Despite this, as there is no longer free access to Repbase, the library used by PASTEC has become outdated. REPCLASS employs a similar strategy alongside structure-based procedures, but their software has not been updated in at least 8 years, and has WU-blast, a discontinued program, as a dependency. RepeatClassifier (installed with RepeatModeler) can use Dfam as the database for its classification task, circumventing the challenge of accessing up-to-date data from Repbase. However, the output of this software is very streamlined, consisting only of a multi-fasta file containing the TE classification in the original sequence header.

While all three of these tools are designed to categorize TEs of any kind, some tools concentrate on doing so in greater detail. Both TEsorter and RTclass1 can classify LTRs and LINEs at the clade level. RTclass1, a Repbase database service, can classify TE at the clade level in seconds; the user only needs to supply the amino acid sequence of the TE protein’s reverse transcriptase domain. Despite being easy, it only works for non-LTR TEs. TEsorter, like most TE-related programs, requires a local installation; however, it is quite simple to install using the conda package manager. This software compares translated TE sequences to profiles in GypsyDB and RexDB. However, while it can generate a classification for any type of TE, it can only classify LTR-type TEs at the clade level. TEclass was one of the first classifiers to use machine learning algorithms. It was last updated in 2016, when the Random Forest and LVQ algorithms were added to the SVM algorithm that had previously been used in the classifier’s first version. In addition to the local installation option, it also provides the option to run the analyses on a web server, making it easier to use for less experienced users. Despite this, the program can only classify TEs into one of four major groups: DNA, LINE, LTR or SINE.

This limitation was recently overcome by DeepTE, ClassifyTE, RFSB and TERL, which also use machine learning (usually artificial neural networks) to classify TEs at the superfamily level. These four programs all run only locally, requiring installation, which may be difficult for some users. Another issue that three of these programs have in common is that they all generate only one classification label for each sequence, even though their output structures differ, only RFSB returns a matrix containing probabilities for each label. TERL, for example, replaces a sequence’s entire header with its classification label, making it difficult for the user to manage multi-fasta files and track back the TEs copies to specific genomic loci. There is also no information about the accuracy of each class prediction in any of these three programs. Machine-learning based tools usually use a k-mer or one-hot encoding approach, which does not take into consideration sequence context or other sequence structural features. Furthermore, other factors can have an impact on the user experience. For example, ClassifyTE requires that the TE library that needs to be classified be located in the “data’’ folder in the application’s root directory, which can limit the application’s flexibility.

## Discussion

In the quest to better understand and unravel the complexity of life from a genomic perspective, bioinformatics has become an indispensable ally of geneticists and molecular biologists. The exponential availability of genomic datasets creates an increasing demand for the development of tools capable of balancing efficiency and ease of use, preventing either from becoming a hindrance to research. Because of a plethora of genetic and structural features that make correct annotation difficult, TEs add another dimension to this picture. To undertake such hardships, many strategies are employed to detect and characterize TEs on genomes.

In the first section of the discussion called ‘the good and the bad’, we present what are the strongest features, *i.e.*, the advantages of using the said method, and what points it may be not so good, interfering in the accuracy of the result. Next, in the section called ‘And the “ugly” side’, we discuss the development status of some of the current tools available, of their documentation, why the ugly side matters, how it impacts research, and we reinforce the arguments on an already existing discussion, although often overlooked, on how to improve on this topic. 

## The good and the bad

Similarity-based tools (RepeatMasker, CENSOR) employ a well-established method that uses libraries or sets of known sequences that for an increasing number of species have experimental validation, generating precise results. The bad side is that it depends on the reliability of the dataset used as a library, its efficiency and precision can quickly decrease when used to detect, for example, protein sequences with only a few distinct residues, and it is time demanding and memory consuming (Zielezinski *et al.*, 2017). 

Structure-based methods, such as LTR_FINDER and MITE-hunter, are best-tailored to detect protein domains or class-specific patterns of TE sequences. The search strategy behind structure-based methods is either an enumerative approach, where sequences are analyzed as small words contained in the query and then compared to a collection of patterns, or probabilistic, in which patterns are searched using a motif or a weighted matrix (Hashim *et al.*, 2019). Equally to similarity-based tools, the search time increases as the dataset grows and is also dependent on known patterns. Nonetheless, when compared to the amount of TE libraries for use with similarity-based tools, there are even less structures/motifs available.

RepeatModeler and EDTA, for example, use the *de novo* methodology to annotate TEs, which is effective for discovering novel TE sequences and creating a non-redundant TE collection. Most of the time, *de novo* tools operate by automatically comparing sequences and grouping those that share the most similarities (Storer *et al.*, 2022). The disadvantage of this method is that it produces more false-positive results than other approaches, is more likely to result in chimeric sequences, and may make it more difficult to distinguish between different TE fragments, sometimes even including pieces of non-TE sequences like those from repetitive gene families from the host genome.

Combining strategies is currently a scientific consensus as a way to minimize the drawbacks of a technique while maximizing its benefits (Arkhipova, 2017). Nonetheless, combining methods brings its own problems to the game. Combining methods also entails combining the disparate output of each program, analyzing the results, removing redundant but not necessarily identical TE sequences, and typically clustering the results. All of this takes more time and computational resources to run, and it does not solve the problem of redundant sequences being classified with different labels. That is why understanding how each method works, as well as the benefits and drawbacks of the tools used, is critical to knowing what results to expect from the annotation.

## And the “ugly” side

Regardless of the good and bad of each software’s methodology, if it is unclear what is needed to install it, how to use it, and how comprehensible the output is, the researcher may opt to avoid using cutting-edge or more performant softwares in favor of older but better documented tools. In other words, when annotating TEs, or even in bioinformatics in general, user friendliness and documentation completeness must be considered.

A poorly documented software may lead the daily work of a researcher to setbacks and delays, by adding a new layer of complexity to the already complex task of working with biological data (Lawlor and Sleator, 2020). It would be similar to conducting a wet lab experiment without fully understanding the chemicals, their activities, or not having the label’s information regarding concentration. It is especially true for small research groups or underfunded institutions that do not have enough financial support to hire a specialist to work on the task, which can become, at a certain level, an obstacle to progress in their field of study and to keep pace with the state-of-the-art (Krampis, 2022). If the quality of software documentation was evaluated as carefully as other topics in peer-reviewed papers on bioinformatics tools, it could contribute to better documented software. Karimzadeh and Hoffman (2018) propose guidelines for creating good software documentation, including, as minimum requirements, a page with code and an issue tracker (*e.g*. Github and Gitlab), a “Readme” file containing the main points for installation and usage, and a manual with a detailed description of every parameter.

It is not uncommon for TE annotation software to use discontinued or outdated packages, causing installation and usage issues, as well as becoming a bottleneck to computer performance, which goes against the ever-increasing computer power and technological advances in operating systems and programming languages. It may also occur as a result of the software’s development being halted and becoming an *abandonware*, not receiving any upgrades, also affecting the developer’s error support for users. Another issue is retro-fitting older tools to new conditions, *i.e.*, a tool developed to identify a certain feature may be unable to extract all the correct information obtained by newer research leading to incomplete results. (Lawlor and Walsh, 2015). The software installation can be impacted by outdated packages, whether it is because the required program does not have a version for more recent operating systems, or because the software’s dependencies cannot be installed. Attempting to install outdated versions on newer platforms may result in version conflicts, leading to the “dependency hell,” a frustrating situation in which a software cannot be utilized due to incompatibilities between software with shared packages that need different versions, particularly for software that require a large number of packages.

Virtual environments and containers are methods for dealing with dependency issues, allowing programs to run on any system (Krampis, 2022). Version conflicts can, however, still occur in virtual environments such as Conda. Containers are more reliable in this regard because they provide a more isolated environment due to operating system-level virtualization, but may be trickier to set up. Dockerfiles and Conda recipes, files containing all commands and software versions to automatically assemble a container or create a virtual environment, make software installation easier, aid in experiment reproducibility, and avoid dealing with dependency issues that may arise when manually installing software and looking for its dependencies.

**Figure 2 -:**
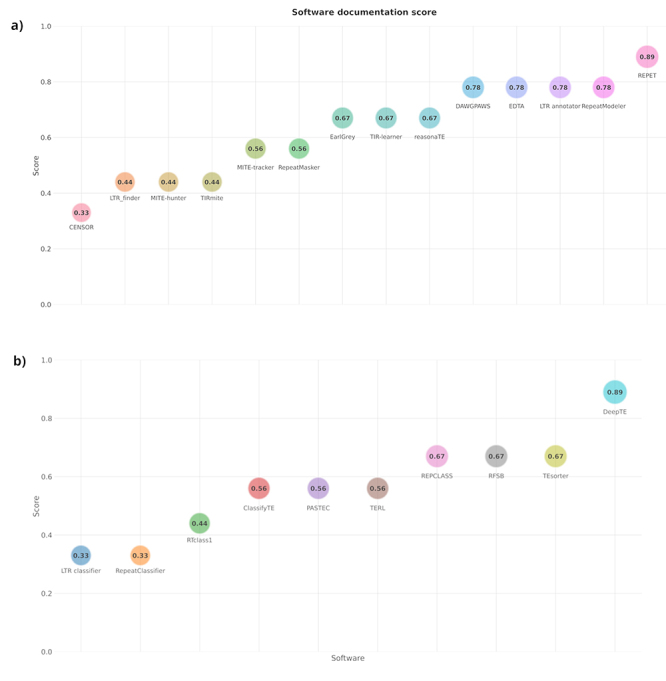
Software score based on documentation availability. The final score, which ranges from 0 to 1, is determined by the presence or absence of various types of documentation, such as a manuscript, reference manual, Readme file, quick start section, informative figure demonstrating how the software works, frequently asked questions (FAQ), news section, issue tracker, and built-in help.

Lack of documentation, software updates and developer support are examples of the “ugly side” of TEs annotation tools and bioinformatics as a whole, all of which are unrelated to the good and bad of each method. On top of that, the ugly side may enter the picture when a developer creates a program to solve his/her own research problem, releases it for the scientific community, but does not fully adapt it for general use, casting aside good software development practices for what worked on the original project. Parameters and outputs that appear clear to the developer may be confusing to the end-user, making the program less user friendly and less understandable for biologists or other life scientists, who are the best suited to validate the findings (Lerat, 2010). In an ideal world, a biologist would have the skills of a software engineer and vice versa, however this is far from reality due to the complexity of both disciplines. The adoption of best practices for developing and deploying bioinformatics software, along with software documentation that adheres to guidelines to better inform the user, would provide a solid foundation for improving TEs annotation tools and the standard of related research (Lawlor and Walsh, 2015; Lawlor and Sleator, 2020). The creation of better documented and user-friendly tools can be aided by initiatives like TE Hub (Elliott *et al.*, 2021), a collaborative platform that aims to provide information for the TE scientific community with a focus on databases, tools, and methods for TE annotation. TE Hub offers a way to integrate information and standardize protocols for tools related to TE scientific research. Figure 2 depicts a score for the tools mentioned here, based on the availability or absence of several types of documentation, such as a reference manual, an informative figure illustrating how the software works, and whether there is an alternative method of installation other than manual installation. Table S1 contains a more detailed version that shows what features are present or absent for each software.

## Final remarks

When choosing a TE annotation software, researchers should always ask themselves: is this the best tool for my needs? What are the downsides? Is the documentation clear about what is required to use the software? Is the software still being actively developed/maintained? Does the developer provide user support? These questions might seem simple, but given the significance of knowing how to get the most out of a tool, they help to achieve better research results, particularly in terms of software usability. Having a reliable TE annotation is the ultimate goal which can be accomplished by improving the status of existing tools from both end-user and developers sides. For that, the user requires better documented tools, as well as a place to share information with the developer so that the developer knows what to do to further develop a more well-structured tool, benefiting the entire TEs scientific community.

## Supplementary material

The following online material is available for this article:

Table S1 - Presence or absence of types of documentation by software.
