# Supplementary material for: The good, the bad and the ugly of transposable elements annotation tools
Source: Genet Mol Biol. 2024 Feb 19;46(3 Suppl 1):e20230138. doi: 10.1590/1678-4685-GMB-2023-0138 (PMC10876081; doi:10.1590/1678-4685-GMB-2023-0138)
Supplement: Table S1 - [file 1415-4757-GMB-46-03-s1-e20230138-s1.pdf]

Genetics and Molecular Biology
https://doi.org/10.1590/1678-4685-GMB-2023-0138

Supplementary Material to "The good, the bad and the ugly of transposable elements annotation tools"

Table S1 - Presence or absence of types of documentation by software.

Software	Manuscript	Reference Manual	Readme	Quick start	Informative figures	FAQ	News	Issue tracker	Built-in help	Score
CENSOR	1	0	0	0	0	0	1	0	1	0.33
ClassifyTE	1	0	1	1	0	0	0	1	1	0.56
DAWGPAWS	1	1	1	1	0	0	1	1	1	0.78
DeepTE	1	0	1	1	1	1	1	1	1	0.89
EarlGrey	1	0	1	1	1	0	0	1	1	0.67
EDTA	1	1	1	1	1	0	0	1	1	0.78
LTR annotator	1	1	1	1	1	0	1	0	1	0.78
LTR classifier	1	0	0	0	1	0	0	0	1	0.33
LTR_finder	1	0	1	0	0	0	0	1	1	0.44
MITE-hunter	1	1	0	0	0	0	0	1	1	0.44
MITE-tracker	1	0	1	0	0	1	0	1	1	0.56
PASTEC	1	1	1	1	0	0	0	0	1	0.56
reasonaTE	1	0	1	1	1	0	0	1	1	0.67
REPCLASS	1	0	1	1	0	0	1	1	1	0.67
RepeatClassifie r	1	0	0	0	0	0	0	1	1	0.33
RepeatMasker	0	0	1	0	0	1	1	1	1	0.56
RepeatModeler	1	1	1	1	0	0	1	1	1	0.78
REPET	1	1	1	0	1	1	1	1	1	0.89
RFSB	1	0	1	1	1	0	0	1	1	0.67
RTclass1	1	0	0	0	1	0	1	0	1	0.44
TERL	1	0	1	1	0	0	0	1	1	0.56
TEsorter	1	0	1	1	1	0	0	1	1	0.67
TIR-learner	1	0	1	1	1	0	0	1	1	0.67
TIRmite	0	0	1	1	0	0	0	1	1	0.44

Legend

0	The absence of a feature.
1	The presence of a feature.
Manuscript	Conceptual and technical details of the method.
Reference manual	Complete details of every configurable setting, input and output.
Readme	Basic instructions for installation and use of the software and where to find more information. Describe how to install your software and all of its dependencies, in detail.
Quick start	Step-by-step instructions for installation and use of the software on a provided test data set, tells users exactly how to get a result with a small number of explicit steps on a specified test data set.
Informative figures	A schema that explains how the software works, and its modules.

News	Changes in behavior, bug fixes, new features and caveats.
FAQ	Answers to commonly asked or anticipated questions.
Issue tracker	News and discussion of details not otherwise provided in the documentation or not apparent to users. A chanel where users can send questions and feedback, ex: GitHub Issues.
Built-in help	Concise description of a software component and its parameters.
Score	A value between 0 and 1, which is the sum of each feature value divided by the number of features.
